# Towards Scalable Probabilistic Linkage of Administrative Population Data in Germany: Insights from a Method Test with Splink

**DOI:** 10.23889/ijpds.v11i4.3814

**Published:** 2026-07-06

**Authors:** René Kramer

**Affiliations:** 1 Destatis, Wiesbaden, Germany

Linking administrative population data is becoming increasingly important for official statistics, yet it remains particularly challenging in Germany due to decentralized data structures, heterogeneous data formats, the absence of a unique identifier, and the sheer volume of potential record comparisons. This contribution presents key methodological considerations and practical lessons from a method test conducted at the Federal Statistical Office of Germany in preparation for future census-related linkage tasks.

The study outlines essential preparatory steps, including variable standardization and normalization, as well as the selection of appropriate linkage strategies. It contrasts exact and deterministic approaches with probabilistic record linkage and highlights the advantages of the latter in the presence of typographical errors, missing values, and inconsistencies across registers. Particular attention is given to the use of Splink, an open-source Python library implementing the Fellegi-Sunter framework in scalable environments such as Spark. Its support for blocking, similarity-based comparison, match weighting, and clerical review makes it a promising tool for large-scale administrative linkage.

Initial findings from the method test indicate that probabilistic linkage provides a flexible and scalable framework for identifying plausible matches. These results underline the potential of modern linkage methods for official population statistics in complex administrative settings.

